# Health Related Behaviours in Normal Weight and Overweight Preschoolers of a Large Pan-European Sample: The ToyBox-Study

**DOI:** 10.1371/journal.pone.0150580

**Published:** 2016-03-07

**Authors:** Greet Cardon, Ilse De Bourdeaudhuij, Violeta Iotova, Julie Latomme, Piotr Socha, Berthold Koletzko, Luis Moreno, Yannis Manios, Odysseas Androutsos, Marieke De Craemer, ToyBox-study group

**Affiliations:** 1 Ghent University, Ghent, Belgium; 2 Medical University Varna, Varna, Bulgaria; 3 Children’s Memorial Health Institute, Warsaw, Poland; 4 Ludwig-Maximilians-University of Munich, Dr. von Hauner Children’s Hospital, Munich, Germany; 5 University of Zaragoza, Zaragoza, Spain; 6 Harokopio University, School of Health Science & Education, Department of Nutrition-Dietetics, Athens, Greece; Vanderbilt University, UNITED STATES

## Abstract

The aim of this study was to investigate the associations of health related behaviours (HRB) with Body Mass Index (BMI) in preschoolers, and to study the likelihood of being overweight/obese in relation to compliance with recommended HRB. The sample consisted of 3301 normal weight and overweight/obese preschoolers (mean age: 4.7 years; 52% boys, 85% normal weight) from six European countries (Belgium, Bulgaria, Germany, Greece, Poland, Spain). Height and weight were measured, total daily step counts were registered during six days, and HRB were assessed with validated parental surveys in 2012. Multiple linear and logistic regression analyses were performed. Only few HRB were significantly associated with BMI. In boys, higher water intake and higher soft drink and higher fruit consumption were significantly associated with higher BMI. Boys drinking less water than recommended were less likely to be overweight/obese (OR = 0.60), while boys who consume soft drinks were more likely to be overweight/obese (OR = 1.52). In girls, higher water intake, higher vegetable consumption, and more TV time on weekend days were significantly associated with higher BMI. Girls eating less vegetables than recommended were less likely to be overweight/obese (OR = 0.62), and girls who engaged in quiet play for more than 90 minutes on weekend days were more likely to be overweight/obese (OR = 1.64). In general, the associations between HRB and BMI or being overweight/obese were limited and mainly related to dietary intake. Awareness campaigns for caregivers should stress that HRB of young children are important and independent of children’s weight status.

## Introduction

Worldwide, the number of overweight children has increased substantially over the last decades [1]. According to recent surveillances in European children under the age of ten, the prevalence of overweight is 21% in girls and 19% in boys [2]. Childhood overweight and obesity are linked to a wide range of co-morbidities and various psychosocial conditions and has been shown to increase the likelihood of being obese in later childhood [3], and even to track into adulthood in one-third to one-half of cases [4]. Consequently, the prevention of overweight and obesity at young age is of public health interest.

The preschool years have been identified as a crucial time period for childhood obesity prevention, as it is a time period in which energy balance-related behaviours (e.g., dietary intake and physical activity) are established [5], and it is also a period immediately preceding the upswing in Body Mass Index (BMI), known as adiposity rebound [6]. However, the evidence base for effective obesity prevention programs in preschool-aged children is still emerging, and more information is required to better inform prevention program development. It is yet unclear which behaviours are associated with being overweight/obese and which are most prevalent in preschool-aged children.

Several studies reported associations between energy balance-related behaviours and overweight in young children, including physical inactivity [7, 8], excessive screen time [9–11] and excessive consumption of energy dense nutrient poor foods [11, 12]. A recent review on the association of health related behaviours (HRB) with overweight and obesity in preschool children reported strong evidence for a negative association between total physical activity and overweight, and moderate evidence for a positive association between television viewing and overweight [13]. Due to the heterogeneity of the dietary intake indicators, insufficient evidence was found for an association between (specific) dietary behaviours and being overweight.

As HRB may vary substantially between countries, it is of interest to study these behaviours in relation to weight status in a large sample of different European countries. Van Stralen et al. (2012) pooled data of six European studies to identify HRB associated with overweight and obesity [14]. Positive associations between sedentary behaviours and overweight indices were found, whereas physical activity and dietary behaviours were not associated with weight status. However, large differences in design, measured behaviours and measurement instruments, and the inclusion of outdated data in some countries, resulted in limited generalizability of the findings.

To conclude, to better inform obesity prevention efforts in the preschool years, there is a need for studies looking into the associations between HRB and weight indices in preschool children, making use of valid and reliable (preferably objective) measures, including specific sub-behaviours for diet, physical activity and sedentary behaviour in a large cross-national sample. Furthermore, it is of high interest for program development (e.g., communications with parents) to know whether preschoolers who do not comply with current recommendations for HRB have an increased likelihood of being overweight/obese at preschool age.

Consequently, we aimed to study the associations between HRB and BMI in a large sample of preschool children, from six European countries, making use of the same standardized protocol in the entire sample. Including a large pan-European sample and controlling for country differences allows presenting more robust and generalizable findings. Secondly, we aimed to study if children not complying with the recommendations for HRB, were more likely to be overweight/obese compared to those complying with the recommendations. The first study aim allows good comparison with the literature as it is the most common approach to study the association between HRB and weight status. Furthermore it does not suffer shortcomings related to dichotomizing HRB data [15]. The second study aim gives complementary information of high relevance for health promotion efforts. Since overweight rates and several HRB are found to differ between boys and girls already at preschool age [11, 15], analyses were performed for boys and girls separately.

## Methods

### Participants

Participants in the present study were part of a large cross-sectional survey within the European ToyBox-study (www.toybox-study.eu). A detailed description of the ToyBox-study, and a comprehensive description of the design and procedures were published elsewhere [16, 17]. In brief, the ToyBox-study aimed to prevent overweight and obesity in four- to six-year-old children, by developing a multidisciplinary intervention and testing it in six European countries: Belgium, Bulgaria, Germany, Greece, Poland, and Spain. Data on the prevalence of overweight and obesity, HRB and their correlates were collected. The children and their families were recruited at kindergartens, daycare centres or preschool settings, depending on the country regulations and legislation. To enhance clarity, all these settings are referred to as "kindergartens" in the present paper.

Per country, kindergartens from different socio-demographic backgrounds were selected from one or two provinces. After excluding the 20% smallest kindergartens, kindergartens were randomly selected within the municipalities. The ToyBox-study aimed for a minimum sample of 1100 preschoolers and one parent/caregiver per child per country. Before the onset of the study, study approval was obtained by Ethical Committees in all six European countries, in line with national regulations (i.e., the Ethical Committee of Ghent University Hospital (Belgium), Committee for the Ethics of the Scientific Studies (KENI) at the Medical University of Varna (Bulgaria), Ethikkommission der Ludwig- Maximilians-Universität München (Germany), the Ethics Committee of Harokopio University of Athens (Greece), Ethical Committee of Children’s Memorial Health Institute (Poland), and CEICA (Comité Ético de Investigación Clínica de Aragón (Spain)). Furthermore, in line with the procedures, approved by the six Ethical Committees, preschoolers’ parents/caregivers provided written informed consent before being enrolled in the study.

**Fig 1:**
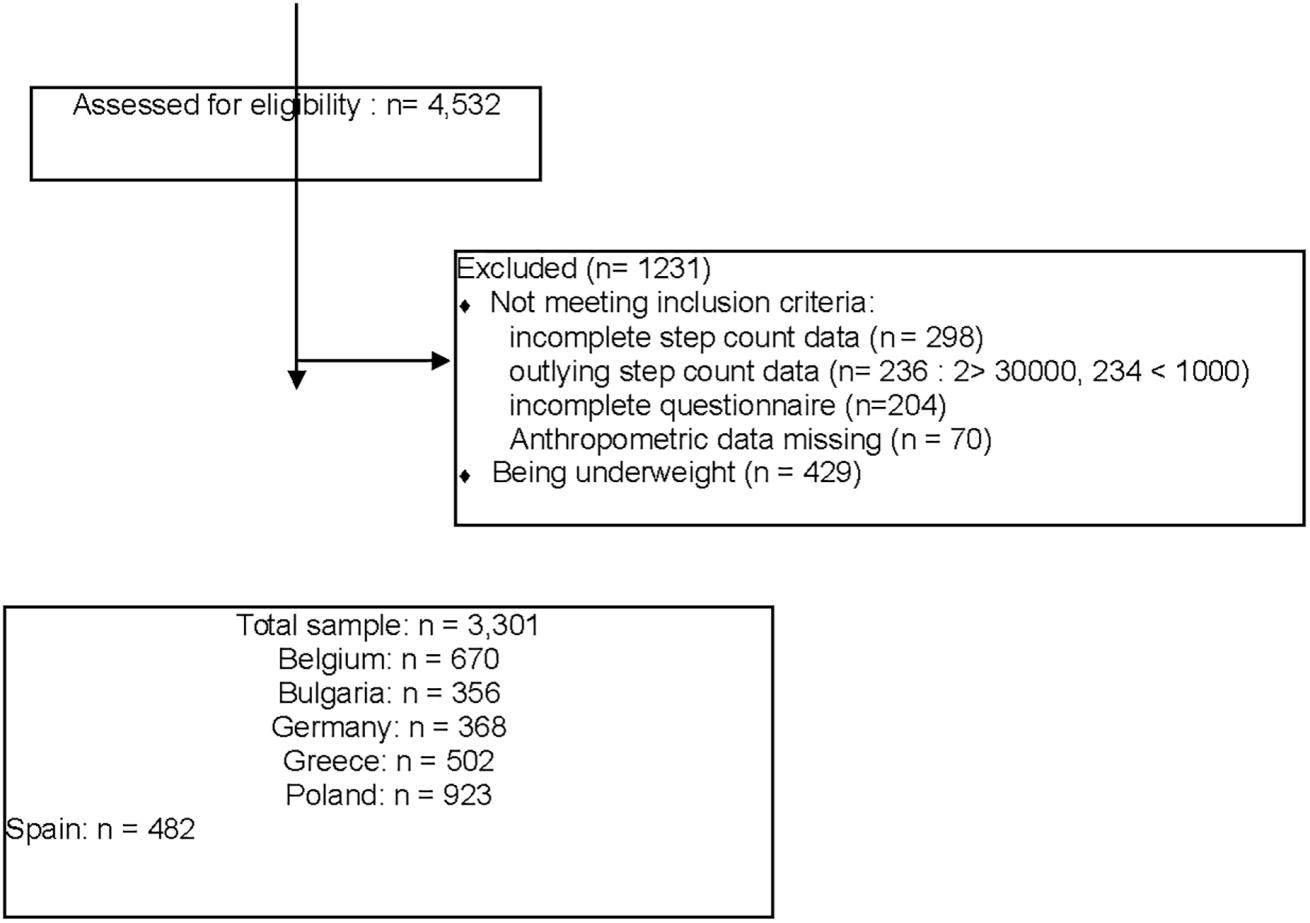
Participants’ flow with exclusion processes.

Data for the present study were collected as baseline measurements of the ToyBox-intervention study in May and June 2012. The randomization of the recruited municipalities to intervention and control group for the ToyBox-intervention study was conducted centrally by the coordinating center (Harokopio University of Athens), after the completion of the baseline measurements. The ToyBox-intervention started after the school summer break, in September 2012. In total, 4532 preschoolers were eligible for all measurements of the present study. After exclusion for missing or outlying step count data, for missing anthropometric data and for incomplete questionnaires a sample of 3730 preschoolers (82%) had complete data for the present study. From this sample, underweight children (11.6% of the boys, 10.9% of the girls) were excluded as correlates of this weight status may be different and specific program development (e.g., for parents of picky eaters) may be preferable [18]. The participants’ flow with reasons for exclusion can be found on Fig 1. Analyses of sample characteristics of excluded compared to included children showed no differences in age (p = 0.95; OR = 0.99; CI = 0.832–1.188), weight status (p = 0.40; OR = 0.90; CI = 0.707–1.147) and gender (p = 0.66; OR = 0.96; CI = 0.803–1.148). However high SES preschoolers had a somewhat higher chance to be included (p = 0.04; OR = 1.208; CI = 1.007–1.450). For the final sample (1716 boys and 1585 girls), distribution over the countries, age, educational level of the mother, BMI and weight status, can be found in Table 1.

**Table 1:** Overview of the sample distribution over the countries. Age, educational level of the mother, BMI and percentages according to weight status in boys and girls. Europe, 2012.

	Boys	Girls
Belgium (n)	351	319
Bulgaria (n)	173	183
Germany (n)	186	182
Greece (n)	250	252
Poland (n)	495	428
Spain (n)	261	221
Total sample (n)	**1716**	**1585**
Age (years)	4.75 ± 0.43	4.76 ± 0.43
Low educational level of the mother (< 14 years of education) (%)	35.7	36.6
High educational level of the mother (≥ 14 years of education) (%)	64.3	63.4
BMI	16.1 ± 1.3	16.1 ± 1.4
Normal weight (%)	86.5	83.3
Overweight (%)	11.0	13.1
Obese (%)	2.6	3.7

### Procedures and measurements

Body height and weight were measured at the kindergartens by trained research assistants according to standardized protocols [17]. Detailed information regarding the procedures and training of research staff and validity and reliability of the measurements were published elsewhere [16, 17, 19, 20]. Children were measured in light clothing without shoes. Body height (Inter-observer reliability: 98,1%) was measured with the SECA 225 Leicester Portable stadiometer (accuracy of 0.1 cm). Weight (Inter-observer reliability: 99,9%) was measured with a calibrated electronic scale SECA 861 (accuracy of 0.1 kg). Of each measurement two readings were obtained and the mean was used for analyses. When the two readings differed by more than 1%, a third measurement was conducted and the mean of the two least deferring values was used. BMI was calculated as weight/height^2^ (kg/m^2^). Weight status (underweight, normal weight, overweight, obese) was obtained according to the International Obesity Task Force thresholds [21].

Based on a literature review on energy-balance-related behaviours in association with overweight and obesity in preschool children [13], a wide range of HRB was included, namely: water, soft drink and sugared milk consumption, fresh fruit and vegetable intake, unhealthy snacking, eating breakfast, daily step counts, outdoor play, screen time and quiet play. Furthermore, as evidence of an association between sleep and childhood obesity is emerging [22–24], sleep duration was also included.

To objectively estimate physical activity levels, total daily step counts were derived from motion sensors. In Bulgaria, Germany, Greece, Poland, and Spain, Omron Walking Style Pro pedometers (HJ-720IT-E2) were used, whereas in Belgium, children were mounted with GT1M or GT3X(+) accelerometers. The Omron Walking Style Pro pedometer and the accelerometer step count function were found to be valid to measure step counts in preschool children (Validation against Actigraph accelerometer counts: pedometer-based step counts: r = 0,64; accelerometer-based step counts: r = 0,89) [25]. Motion sensors were worn on the right hip, secured by an elastic waist band. Preschoolers were asked to wear the motion sensor for six consecutive days, including two weekend days. Preschoolers’ parents, who consented, received an information letter and were asked to let their child wear the measurement device during all waking hours and to remove it only during water-based activities. Pedometer data were downloaded using Omron Health Management software version E1.012, and accelerometer step counts were downloaded using the ActiLife 5.5.5. software. The first (i.e., day of mounting) and sixth day (i.e., day of collection) were omitted, because these days were incomplete. Furthermore, all pedometer—and accelerometer based step counts below 1,000 and above 30,000 steps per day were deleted and treated as missing data since these values are considered outliers [26]. Mean steps per day were separately calculated for weekdays and weekend days.

The children received two parental questionnaires (i.e., the Food Frequency Questionnaire and the Primary Caregivers’ Questionnaire) in a closed envelop to take home for completion by one of the parents/caregivers. The questionnaires can be found online (www.toybox-study.eu) and detailed information on the development, test-retest reliability and validity were described elsewhere [17, 20, 27]. Furthermore an overview of the questions and response options used for the present study can be found in Table 2. Data from the Food Frequency Questionnaire were used for the assessment of the dietary intake variables. The Food Frequency Questionnaire showed moderate to good reliability and acceptable validity against the 3-day-food diary [27]. The Primary Caregivers’ Questionnaire was used for demographics and for data related to outdoor play, sedentary behaviours and sleep duration. Findings showed that 92% of the ToyBox Primary Caregiver Questionnaire had a moderate-to-excellent test—retest reliability (defined as ICC values from 0.41 to 1) and less than 8% poor test—retest reliability (ICC < 0.40) [20]. The questions used for the present study are outlined in Table 2 and are described below.

**Table 2:** Overview of the HRB-items, questions or measurement method, response options, and dichotomisation values.

			Dichotomisation
**Dietary intake**			
**Water consumption** ml/day (1 item)	**How often does the child consume water?** 1. Never or less than once per month 2. 1–3 days per month 3. 1 day per week 4. 2–4 days per week 5. 5–6 days per week 6. Every day	**and which is the AVERAGE AMOUNT PER DAY?** ^1^ 1. 100ml or less 2. Between 100 and 200ml 3. Between 200 and 300ml 4. Between 300 and 400ml 5. Between 400 and 500ml 6. Between 500 and 600ml 7. Between 600 and 700ml 8. Between 700 and 800ml 9. Between 800 and 900ml 10. Between 900 and 1000ml 11. 1000ml or more	0:≥500ml /day 1:<500ml/day
**Soft drink consumption** ml/day (1 item)	**How often does the child consume soft drinks?** 1. Never or less than once per month 2. 1–3 days per month 3. 1 day per week 4. 2–4 days per week 5. 5–6 days per week 6. Every day	**and which is the AVERAGE AMOUNT PER DAY?** ^1^ 1. 100ml or less 2. Between 100 and 200ml 3. Between 200 and 300ml 4. Between 300 and 400ml 5. Between 400 and 500ml 6. Between 500 and 600ml 7. Between 600 and 700ml 8. Between 700 and 800ml 9. Between 800 and 900ml 10. Between 900 and 1000ml 11. 1000ml or more	0: no 1: any
**Sugared milk consumption** ml/day (1 time)	**How often does the child consume sugared milk?** 1.never or less than once a month 2. 1–3 days a month 3. 1 day a week 4. 2–4 days a week 5.5–6 days a week 6. Every day	**and which is the AVERAGE AMOUNT PER DAY?** ^1^ 1. 100 ml or less 2. between 100 and 200 ml 3. between 200 and 300 ml 4. between 300 and 400 ml 5. between 400 and 500 ml 6. Between 500 and 600 ml 7. between 600 and 700 ml 8. between 700 and 800 ml. 9.between 800 and 900 ml 10. between 900 and 1000 ml 11.1000 ml or more	0: no 1: any
**Fresh fruit consumption** (g/day) (1 item)	**How often does the child consume fresh fruit?** 1. never or less than once per month 2.1–3 days per month 3. 1 day per week 4. 2–4 days per week 5. 5–6 days per week 6. every day	**and which is the AVERAGE AMOUNT PER DAY?** ^1^ 1. 30 g or less 2. between 30 and 60 g 3. between 60 and 90 g 4. between 90 and 120 g 5. between 120 and 150 g 6. between 150 and 180 g 7. between 180 and 210 g 8. between 210 and 240 g 9. between 240 and 270 g 10. 270 g or more	0:≥100g/day 1:<100g/day
**Vegetable consumption** (g/day) (2 items: raw vegetables, cooked vegetables)	**e.g. How often does the child consume fresh vegetables?** 1. never or less than once per month 2. 1–3 days per month 3. 1 day per week 4. 2–4 days per week 5. 5–6 days per week 6. every day	**and which is the AVERAGE AMOUNT PER DAY?** ^1^ 1. 30 g or less 2.between 30 and 60 g 3. between 60 and 90 g 4. between 90 and 120 g 5. between 120 and 150 g 6. between 150 and 180 g 7. between 180 and 210 g 8. between 210 and 240 g 9. between 240 and 270 g 10. 270 g or more	0:≥100g/day 1:<100g/day
**Unhealthy snacks** (g/day)(6 items: Chocolate and candy bars, salty snacks, cakes, milk-based desserts, sugar-based desserts,)	**e.g. How often does the child consume chocolate and candy bars?** 1. Never or less than once per month 2. 1–3 days per month 3. 1 day per week 4. 2–4 days per week 5. 5–6 days per week 6. Every day	**and which is the AVERAGE AMOUNT PER DAY?** ^1^ e.g. Chocolate and candy bars: 1. 25g or less 2. Between 25 and 50g 3. Between 50 and 75g 4. Between 75 and 100g 5. Between 100 and 125g 6.125g or more	0:<47mg/day 1:≥47mg/day
**Breakfast consumption** (times/week) Note. A drink alone can not be considered as a meal.	**How often does your child consume breakfast?**	1. (almost) never 2. 1–3 times per month 3. 1 day a week 4. 2–4 days a week 5. 5–6 days a week 6. Every day	0: daily 1: Not daily
**Physical activity**			
**Daily steps on weekdays**	Accelerometer- or pedometer derived step counts		0:≥11500 steps/day 1:<11500 steps/day
**Daily steps on weekend days**	Accelerometer- or pedometer derived step counts		0:≥11500 steps/day 1:<11500 steps /day
**Outdoors play on weekdays** (hours/day)	**How much time did your child spend outdoors in active play yesterday?** If yesterday was a Saturday or Sunday, this question refers to the last weekday.	__ hours __ minutes	0:≥2 hours/day 1:<2hours/day
**Outdoors play on weekend days** (hours/day)	**How much time did your child spend outdoors in active play in the last weekend day?**	__ hours __ minutes	0:≥4 hours/day 1:<4hours/day
**Sedentary behaviour**			
**TV time on weekdays** (min/day)	**About how many hours a day does your child usually watch television (including DVDs and videos) in his/her free time? On weekdays**	1. Never 2. Less than 30 minutes/day 3. 30 minutes to <1 hr/day 4. 1–2 hrs/ day 5. 3–4 hrs/ day 6. 5–6 hrs/ day 7. 7–8 hrs/ day 8. 8 hrs/ day 9. More than 8 hrs/ day	Sum of TV and computer time: 0:<1hour/day 1:≥1hour/day
**Computer use on weekdays** (min/day)	**About how many hours a day does your child use the computer for activities like playing games on a computer, game consoles (e.g., Playstation, Xbox, GameCube) during leisure time? On weekdays**	1. Never 2. Less than 30 minutes/day 3. 30 minutes to <1 hr/day 4. 1–2 hrs/ day 5. 3–4 hrs/ day 6. 5–6 hrs/ day 7. 7–8 hrs/ day 8. 8 hrs/ day 9. More than 8 hrs/ day	Sum of TV and computer time: 0:<1hour/day 1:≥1hour/day
**TV time on weekend days** (min/day)	**About how many hours a day does your child usually watch television (including DVDs and videos) in his/her free time? On weekend days**	1. Never 2. Less than 30 minutes/day 3. 30 minutes to <1 hr/day 4. 1–2 hrs/ day 5. 3–4 hrs/ day 6. 5–6 hrs/ day 7. 7–8 hrs/ day 8. 8 hrs/ day 9. More than 8 hrs/ day	Sum of TV and computer time: 0:<1hour/day 1:1hour/day
**Computer use on weekend days** (min/day)	**About how many hours a day does your child use the computer for activities like playing games on a computer, game consoles (e.g., Playstation, Xbox, GameCube) during leisure time? On weekend days**	1. Never 2. Less than 30 minutes/day 3. 30 minutes to <1 hr/day 4. 1–2 hrs/ day 5. 3–4 hrs/ day 6. 5–6 hrs/ day 7. 7–8 hrs/ day 8. 8 hrs/ day 9. More than 8 hrs/ day	Sum of TV and computer time: 0:<1hour/day 1:1hour/day
**Quiet play on weekdays** (min/day)	**About how many hours a day does your child have quiet play ((looking into books, playing with blocks, playing with dolls, drawing, construction) during leisure time? On weekdays**	1. never 2. Less than 30 minutes/day 3. 30 minutes to one hour/day 4. 1–2 hours/day 5. 3–4 hours /day 6. 5–6 hours/day 7. 7–8 hours/day 8. More than 8 hours/day 9. I don’t know	0:<90 min/day 1:≥90min/day
**Quiet play on weekend days** (min/day)	**About how many hours a day does your child have quiet play (looking into books, playing with blocks, playing with dolls, drawing, construction) during leisure time? On weekend days**	1. never 2. Less than 30 minutes/day 3. 30 minutes to one hour/day 4. 1–2 hours/day 5. 3–4 hours /day 6. 5–6 hours/day 7. 7–8 hours/day 8. More than 8 hours/day 9. I don’t know	0:90min/day 1:≥90min/day
**Sleep duration**			
**Sleep duration at night on weekdays** (hours/night)	**How many hours of sleep does your child usually have during the night? On weekdays**	1. Less than 6 hours 2. 6–7 hours 3. 8–9 hours 4.10–11 hours 5. 12–13 hours 6. 14 hours 7. More than 14 hours 8. I don’t know	0:0≥11 hours/night 1:<11 hours/night
**Sleep duration at night on weekend days** (hours/night)	**How many hours of sleep does your child usually have during the night? On weekend days**	1. Less than 6 hours 2. 6–7 hours 3. 8–9 hours 4.10–11 hours 5. 12–13 hours 6. 14 hours 7. More than 14 hours 8. I don’t know	0:0≥11 hours/night 1:<11 hours/night

^1:^ In the questionnaire examples and colour images are provided to facilitate portion size selection.

#### Food Frequency Questionnaire

For the child’s consumption of water (1 item), soft drinks (1 item), sugared milk beverages (1 item), vegetables (2 items), fresh fruits (1 item) and snacks (6 items) questions were two-fold. First, for each item, the frequency of consumption was asked on a six-point scale, ranging from “never or less than once a month” to “every day”. Next, parents were asked to indicate the size category that best fitted the average consumption per day. The response categories varied depending on the food item and a list of common standard measures (e.g., one cup = 225 ml) was given, as well as colour images to facilitate the selection of portion sizes. In the Food Frequency questionnaire no distinction is made between consumption on week—and weekend days. Data were converted into average daily intake values by multiplication of number of days per week and amount per day divided by 7 [27]. Breakfast consumption was assessed by asking on a six-point scale how many days per week (ranging from “(almost) never” to “every day”) children eat breakfast.

#### Primary Caregivers’ Questionnaire

Parents were asked to report the date of birth and gender of their child. The number of years of education of the parent and their spouse/partner was asked on a five-point scale, ranging from “less than seven years” to “more than 16 years”. For the current study, the number of years of education of the mother was used as a proxy measure of family socio-economic status [28, 29]. For ease of interpretation the educational level was dichotomized into lower (14 or fewer years of education) and high (more than 14 years of education) socio-economic status [30].

Parents were asked to report how many minutes or hours per day their child was engaged in active play outdoors during the last weekday and weekend day (Test-retest reliability ICC_week_ = 0.56; ICC_weekend_ = 0.14). For sedentary behaviour, parents were asked to report how much time their child spends watching TV (Test-retest reliability: ICC_week_ = 0.67; ICC_weekend_ = 0.67), playing on a computer or game console (Test-retest reliability ICC_week_ = 0.72; ICC_weekend_ = 0.81) and playing quietly (e.g., drawing, colouring) (Test-retest reliability ICC_week_ = 0.42; ICC_weekend_ = 0.50) for weekdays and weekend days separately, referring to a general week. Answers ranged from”never” to”more than 8 hours per day”, on a 9-point scale. The variables were then recoded into minutes per day. “Never” was recoded into 0 and “more than 8 hours/day” was recoded into 8 hours/day. The other values were recoded using the mid-point method (e.g. 3–4 hours /day was recoded into 3,5 hours/day) [31]. TV viewing and computer use were summed to reflect screen time. Furthermore, parents were asked, for both week and weekend days, how many hours their child usually sleeps during the night, on a seven-point scale, ranging from “less than six hours” to “more than 14 hours” (Test-retest reliability ICC_week_ = 0,68; ICC _weekend_ = 0,69).

### Statistical analyses

SPSS (version 22) was used for all statistical analyses. Continuous data were checked for normal distribution (skewness < 0.70). They were found to be normally distributed. To study the associations between HRB (independent variables) and BMI (dependent variables) multiple linear regressions were used with HRB and BMI as continuous variables.

To study if children who do not comply with the recommendations for HRB (independent variable) were more likely to be overweight/obese (dependent variable), logistic regression models were used (reference group: normal weight). Therefore, HRB data were dichotomized to distinguish between those complying (0) and those not complying (1) with the recommendations for good health. When no clear recommendations could be located in the literature, the median was used as a threshold for dichotomization (see Table 2). Step counts were dichotomized into 0 (≥11,500 steps/day) and 1 (<11,500 steps/day) based on the physical activity guideline of 11,500 steps/day that corresponds to three hours of total physical activity in preschoolers [32]. For active play outdoors, data were dichotomized based on the medians for weekdays and for weekend days (0≥the median; 1<the median). Screen time was dichotomized into 0 (<1 hour daily screen time) and 1 (≥1 hour daily screen time), based on the most generally accepted recommendation, restricting TV viewing time to 1–2 h per day where the lower threshold applies to pre-school children [33, 34]. The median was used to dichotomize the time children spend in sedentary quiet play (0<the median; 1≥the median). Based on Matricciani et al. (2013) and in line with Kovacs et al. (2014), among the various recommendations for sleep duration, we considered the recommendation of 11 hours or more for preschool children to dichotomize sleep duration (0≥11 hours; 1<11 hours) [34, 35].

As food-based dietary guidelines for preschool children only exist on a national basis, we used nutrient recommendations of the Belgian Health Council [36]. These guidelines are very similar to dietary guidelines in other countries, making them applicable to European preschoolers [37]. Water consumption was dichotomized into: 0 (≥500ml per day) and 1 (<500ml per day). Soft drinks and sugared milk consumption were dichotomized into: 0 (no consumption) and 1 (any consumption).

Both fresh fruit and vegetable intake were dichotomized into those reaching the recommendation (0≥100 g/day) and those not reaching (1<100 g/day). For snacking, the median was used to dichotomize into healthy (0<median) and unhealthy (1≥median) snacking behaviour. For breakfast, consumption data were dichotomized into: 0 (eating breakfast daily) and 1 (not eating breakfast daily) based on the recommendation to consume breakfast daily [38].

Analyses were performed separately for boys and girls. As we aimed to present generalizable findings we did not cluster data into countries but included country (6 categories) as covariate, in addition to age and the educational level of the mother (high versus low). The level of statistical significance was set at p < 0.05.

## Results

89.7 percent of the questionnaires was filled out by the mother, 9.3 percent by the father and 1.0 percent by another caregiver (e.g. stepmother, stepfather, grandparent). Tables 3 and 4 present the associations between the HRB and BMI, based on multiple regression models. For ease of interpretation, the mean scores for the HRB in normal weight and overweight/obese boys and girls are also shown. In boys, higher water intake, higher soft drink consumption and higher fruit intake were significantly associated with higher BMI. In girls, higher water intake, more vegetable consumption, and more TV time on weekend days were significantly associated with higher BMI. In boys only age was a significant co-variate (p = 0.05), while in girls only socio-economic status was a significant co-variate (p = 0.02).

**Table 3:** Mean scores for health related behaviours in normal weight (NW) and overweight/obese (OW) boys and results from the Multiple regression analyses of associations between HRB and BMI in boys. Europe, 2012. (Adj. R^2^ = 0.028; F = 2.665; p<0.001).

	Mean (SD) in NW boys	Mean (SD) in OW boys	Beta	t	p	95% CI Lower	95% CI Upper
**Dietary intake**							
Water consumption ml/day	540.88 (314.87)	626.62 (322.92)	0.108	3.642	**<0.001**	0.000	0.001
Soft drink consumption ml/day	69.20 (153.84)	96.68 (185.23)	0.096	3.243	**0.001**	0.000	0.001
Sugared milk consumption ml/day	44.67 (98.27)	50.93 (115.49)	0.031	1.078	0.28	0.000	0.001
Fruit consumption (g/day)	128.22 (71.42)	139.88 (81.36)	0.069	2.243	**0.03**	0.000	0.002
Vegetable consumption (g/day)	105.15 (83.40)	105.71 (88.79)	-0.03	-0.109	0.91	-0.001	0.001
Unhealthy snacks (g/day)	61.03 (47.94)	64.12 (51.88)	0.07	0.236	0.81	-0.001	0.002
Breakfast consumption (times/week)	6.60 (1.34)	6.52 (1.58)	-0.013	-0.441	0.66	-0.059	0.041
**Physical activity**							
Daily steps on weekdays	11137 (3407)	10892 (3639)	0.003	0.107	0.92	0.000	0.000
Daily steps on weekend days	9809 (4202)	9310 (4176)	-0.047	-1.570	0.12	0.000	0.000
Outdoor play on weekdays (hours/day)	2.23 (1.75)	2.15 (1.84)	-0.031	-1.005	0.32	-0.065	0.021
Outdoor play on weekend days (hours/day)	4.42 (3.07)	4.27 (2.87)	0.019	0.602	0.55	-0.014	0.036
**Sedentary behaviour**							
TV time on weekdays (min/day)	65.82 (50.13)	73.73 (52.09)	0.024	0.639	0.52	-0.001	0.003
TV time on weekend days (min/day)	110.88 (77.26)	120.34 (75.92)	0.038	1.027	0.31	0.000	0.002
Computer use on weekdays (min/day)	12.80 (23.68)	20.20 (41.29)	0.025	0.634	0.53	-0.004	0.004
Computer use on weekend days (min/day)	28.02 (43.51)	35.63 (60.80)	0.018	0.457	0.65	-0.001	0.003
Quiet play on weekdays (min/day)	80.39 (65.67)	79.63 (64.19)	-0.001	-0.019	0.99	-0.001	0.001
Quiet play on weekend days (min/day)	122.19 (88.19)	115.47 (85.96)	-0.040	-1.136	0.26	-0.002	0.000
**Sleep duration**							
Sleep duration at night on weekdays (h/night)	9.98 (1.22)	9.87 (1.21)	0.023	0.630	0.53	-0.047	1.030
Sleep duration at night on weekend days (h/night)	10.38 (1.31)	10.19 (1.20)	-0.057	1.633	0.10	-0.130	0.006

Adjusted for country, age, educational level of the mother

**Table 4:** Mean scores for health related behaviours in normal weight (NW) and overweight/obese (OW) girls and results from the Multiple regression analyses of associations between HRB and BMI in girls. Europe, 2012. (Adj. R^2^ = 0.032; F = 2.772; p<0.001).

	Mean (SD) in NW girls	Mean (SD) in OW girls	Beta	t	p	95% CI Lower	95% CI Upper
**Dietary intake**							
Water consumptionml/day	512.70 (304.34)	575.77 (318.10)	0.108	3.446	**0.001**	0.000	0.001
Soft drink consumptionml/day	65.53 (143.04)	68.15 (134.52)	0.025	0.764	0.45	0.000	0.001
Sugared milk consumption ml/day	37.46 (88.47)	29.28 (63.01)	-0.027	-0.903	0.37	-0.001	0.001
Fruit consumption (g/day)	127.83 (71.45)	134.34 (74.50)	-0.025	-0.738	0.46	-0.002	0.001
Vegetable consumption (g/day)	106.27 (81.36)	123.85 (90.86)	0.081	2.363	**0.02**	0.000	0.002
Unhealthy snacks (g/day)	59.26 (49.97)	57.48 (48.86)	-0.037	-1.155	0.25	-0.003	0.001
Breakfast consumption (times/week)	6.51 (1.49)	6.37 (1.61)	-0.021	-0.686	0.49	-0.078	0.029
**Physical activity**							
Daily steps on weekdays	9865 (3012)	9603 (3279)	0.012	0.388	0.70	0.000	0.000
Daily steps on weekend days	8809 (3791)	8652 (3916)	-0.014	-0.439	0.66	0.000	0.000
Outdoor play on weekdays (h/day)	2.06 (1.77)	2.05 (1.72)	0.015	0.459	0.65	-0.035	0.064
Outdoor play weekend days (h/day)	4.19 (3.08)	4.14 (3.07)	-0.011	-0.339	0.735	-0.036	0.020
**Sedentary behaviour**							
TV time on weekdays (min/day)	64.38 (51.48)	72.49 (54.10)	-0.032	-0.805	0.421	-0.003	0.001
TV time on weekend days (min/day)	109.92 (79.25)	124.06 (81.40)	0.145	3.663	**<0.001**	0.001	0.004
Computer use on weekdays (min/day)	9.64 (24.64)	11.50 (21.01)	0.032	0.698	0.49	-0.004	0.008
Computer use on weekend days (min/day)	19.21 (35.93)	20.45 (33.73)	-0.072	-1.576	0.12	-0.006	0.001
Quiet play on weekdays (min/day)	90.73 (67.54)	87.11 (64.75)	-0.007	-0.192	0.85	-0.002	0.001
Quiet play on weekend days (min/day)	144.76 (92.11)	145.28 (95.75)	0.014	0.379	0.71	-0.001	0.001
**Sleep duration**							
Sleep duration at night on weekdays (h/night)	10.05 (1.16)	9.72 (1.25)	-0.015	-0.392	0.70	-0.106	0.067
Sleep duration at night on weekend days (h/night)	10.52 (1.24)	10.24 (1.30)	-0.043	-1.187	0.24	-1.121	0.035

Adjusted for country, age, educational level of the mother

The percentages of boys and girls not complying with the recommendations for the different HRB and the results of the logistic regressions can be found in Tables 5 and 6 for boys and girls, respectively. Boys drinking less water than recommended were less likely to be overweight/obese (p = 0.01; OR = 0.60) and boys who consumed soft drinks were more likely to be overweight/obese (p = 0.02; OR = 1.52). Girls eating less vegetables than recommended were less likely to be overweight/obese (p = 0.01; OR = 0.62), girls who engaged in quiet play for more than 90 minutes on weekend days were more likely to be overweight/obese (p = 0.03; OR = 1.71). Both boys (p = 0.09; OR = 1.62) and girls (p = 0.06; OR = 1.64), sleeping less than 11 hours on weekend days were more likely to be overweight/obese, however for these associations only a trend towards significance was found. In both boys (p = 0.05) and girls (p = 0.04) only socio-economic status was a significant co-variate.

**Table 5:** Percentages of normal weight (NW) and overweight/obese (OW) boys not complying with the recommendations and associations between health related behaviours and weight status in boys—Logistic regression. Europe, 2012.

	NW boys (reference group) %	OW boys %	Odds ratio	P-value	95% CI Lower	95% CI Upper
**Dietary intake**						
Water intake < 500ml/day	46.8	34.5	0.604	**0.01**	0.402	0.868
Soft drink consumption	58.1	62.8	1.516	**0.02**	1.061	2.166
Sugared milk consumption	45.8	46.6	0.997	0.99	0.524	1.081
Fruit intake < 100g/day	34.8	31.3	0.753	0.12	0.524	1.081
Vegetable intake < 100g/day	55.3	58.8	1.220	0.24	0.875	1.700
Unhealthy snacking ≥ 47mg/day	51.0	53.7	1.174	0.34	0.845	1.633
No daily breakfast consumption	10.3	10.1	0.893	0.67	0.525	1.517
**Physical activity**						
< 11500 steps on weekdays	56.6	61.5	1.269	0.17	0.905	1.780
< 11500 steps on weekend days	68.0	71.2	1.036	0.63	0.721	1.488
Less than 2 hours active play outdoors on weekday	37.5	44.8	1.299	0.14	0.917	1.840
Less than 4 hours active play outdoors on weekend day	41.5	46.1	1.060	0.74	0.748	1.501
**Sedentary behaviour**						
≥ 1 hour screen time on weekdays	57.3	64.3	1.099	0.63	0.754	1.601
≥ 1 hour screen time on weekend days	81.9	86.9	1.054	0.84	0.636	1.748
≥ 90 min quiet play on weekdays	53.3	52.4	1.073	0.72	0.727	1.584
≥ 90 min quiet play on weekend days	74.4	71.1	0.993	0.98	0.638	1.547
**Sleep**						
< 11 hours sleep at night on weekdays	93.9	94.4	0.785	0.53	0.370	1.666
< 11 hours sleep at night on weekend days	84.2	89.6	1.618	**0.09**	0.923	2.853

CI: confidence intervals

Adjusted for age, educational level of the mother, and country

**Table 6:** Percentages of normal weight (NW) and overweight/obese (OW) girls not complying with the recommendations and associations between health related behaviours and weight status in girls—Logistic regression. Europe, 2012.

	NW girls (reference group) %	OW girls%	Odds Ratio Sign	P-value	95% CI Lower	95% CI Upper
**Dietary intake**						
Water intake < 500ml/day	50.2	44.0	0.834	0.31	0.587	1.185
Soft drink consumption	54.9	56.2	1.228	0.25	0.866	1.743
Sugared milk consumption	46	40.9	0.815	0.22	0.586	1.133
Fruit intake < 100g/day	34.9	32.4	0.955	0.80	0.667	1.367
Vegetable intake < 100g/day	55.5	47.1	0.623	**0.01**	0.445	0.873
Unhealthy snacking ≥ 47mg/day	49.0	46.0	0.892	0.50	0.642	1.239
No daily Breakfast consumption	12.6	15.9	1.401	0.13	0.901	2.180
**Physical activity**						
< 11500 steps on weekdays	74.1	73.1	0.976	0.89	0.683	1.393
< 11500 steps on weekend days	88.0	76.4	0.826	0.31	0.571	1.194
Less than 2 hours active play outdoors on weekday	41.6	42.3	0.930	0.70	0.663	1.315
Less than 4 hours active play outdoors on weekend day	44.5	44.2	1.057	0.75	0.754	1.481
**Sedentary behaviour**						
≥ 1 hour screen time on weekdays	52.3	59.6	1.152	0.44	0.806	1.647
≥ 1 hour screen time on weekend days	79.2	91.5	1.024	0.92	0.639	1.640
≥ 90 min quiet play on weekdays	61.9	59.4	0.790	0.19	0.557	1.122
≥ 90 min quiet play on weekend days	91.9	93.5	1.715	**0.03**	1.047	2.810
**Sleep**						
< 11 hours of sleep at night on weekdays	93.0	93.9	0.929	0.85	0.429	2.013
< 11 hours of sleep at night on weekend days	91.7	88.0	1.637	**0.06**	0.975	2.750

CI: confidence intervals

Adjusted for age, country and mother’s educational level

## Discussion

In the present study, only limited and weak associations were found between HRB and BMI and the explained variances of the models were very low. In boys, a higher intake of water, soft drinks and fruit were significantly associated with higher BMI. In line with van Stralen et al. (2012), the other dietary intake variables were not associated with BMI [14]. In the present study, physical activity and sedentary behaviour were not significantly associated with BMI in boys. In girls, higher water intake and higher vegetable consumption were associated with higher BMI. In line with Van Stralen et al. (2012), more TV time on weekend days associated with higher BMI in girls, while physical activity variables were not associated with BMI [14]. In the literature, inconsistent results were found on the associations between HRB and weight indices in young children and limited associations have been reported previously [13, 14]. Making use of standardized methods, the present study confirms the limited associations in a large pan-European sample. These limited associations may be due to the fact that an unbalanced diet, low physical activity levels and high sedentary behaviour levels may not impact weight status until a later age. This finding is important in the scope of intervention development for parents, as the time lag between HRB and children’s body composition may result in limited motivation in parents to change practices. Poor HRB per se may not be perceived as problematic, and parents and other caregivers should be sensitized that HRB are important for long term health promotion, regardless of absence of obesity in their preschool child, as these behaviours are already established at preschool age and may result in an unhealthy weight status at a later age. Furthermore, these behaviours are important for a healthy development of the child (e.g., motor development, bone density). The potential time lag between HRB and weight status also favours the use of HRB in addition to weight status as an indicator of the effectiveness of obesity prevention interventions among preschool-aged children. The direct effect of programs and strategies on body composition parameters may not be evident during the preschool years.

In line with the findings of Hardy et al. (2012) in Australian children commencing school, we found that many European children have several poor HRB, even before they start school [11]. For most HRB, only about one half of the normal weight as well as the overweight/obese children did comply with the recommendations, which is in line with Kovacs et al. (2014) [34].

However, we also found that not complying with the recommendations for HRB was associated with a higher likelihood of being overweight/obese for only a few HRB. In the present study, boys who consumed soft drinks were more likely to be overweight, but surprisingly we also found that boys who drank less water than recommended were less likely to be overweight/obese. In girls, non-compliance with the recommendations for water and soft drink consumption did not associate with a higher likelihood to be overweight, but surprisingly girls who consumed less vegetables than recommended were less likely to be overweight/obese.

Given the cross-sectional nature of this study, the positive association between water intake and BMI and the lower likelihood to be overweight/obese in boys drinking less water than recommended can possibly be explained by the fact that the promotion of water intake is a health message that is probably well known in the scope of weight management. Therefore, it can be speculated that parents or other caregivers encourage overweight/obese children to drink more water. This might also be applicable for the finding that girls who eat less vegetables than recommended are less likely to be overweight.

The fact that boys who consume soft drinks are more likely to be overweight/obese than those not consuming soft drinks, emphasizes the need to target the limitation of soft drink consumption in young boys, especially since we found that the soft drink consumption was much higher in overweight/obese boys (97 ml/day) compared to their normal weight peers (69 ml/day). In girls, this difference was much less pronounced.

In boys, not complying with the recommendations for physical activity and sedentary behaviour was not associated with an increased likelihood of being overweight/obese. Again, this may be due to the time lag between HRB and weight impact. Girls who engaged in more than 90 minutes of quiet sedentary play, were more likely to be overweight/obese. To our knowledge, no previous studies looked into this association. However, according to our findings, this sedentary behaviour is very common in preschoolers and deserves targeting in the scope of obesity prevention.

While evidence is emerging that sleep duration is inversely associated with childhood obesity [22–24], the current study only found a marginally significant increased likelihood of being overweight/obese in boys and girls not sleeping a minimum of 11 hours at night on weekend days. A time lag between behaviour and weight status might also be applicable for this finding. Another possible explanation is that the current study results were corrected for the educational level of the mother, so that any association with obesity may be weakened by this adjustment. Speirs et al. (2014) found full-time maternal employment as an important predictor of shortened night-time sleep among preschool children [39]. Furthermore bedtime and wake time data might be of interest to consider next to sleep duration. However these data were not available in the present study.

Given the cross-sectional nature, the present study did not aim to show causal interference between HRB and weight status in preschoolers. However, the associations between a wide range of HRB and BMI and between not complying with HRB recommendations and the likelihood to be overweight/obese were studied. Apart from the inclusion of a large pan-European sample of preschoolers from six countries, the use of standardized methods and the objective measurement of height, weight and step counts throughout the entire sample were strengths of the current study. We acknowledge that the sample included in the present study is not a fully representative sample, due to sampling in specific regions in each country. Moreover selection bias may have occurred due to the exclusion of 27 percent of the initial sample, based on missing or outlying data and due to the fact that only pre-schoolers attending kindergarten were included. However, about 99% of all preschool-aged children attend kindergarten in Belgium, about 98% in Spain, about 95% in Germany, about 70% in Bulgaria, and about 45% in Greece and Poland [40]. Furthermore in the final sample the representation of high and low educational levels of the mothers is typical [41], (almost) complete class groups were included in the kindergartens, the prevalence of overweight and obesity is comparable to previous studies in European pre-schoolers [2, 14] and the sample characteristics of the included and excluded preschoolers did not differ in age, gender and weight status and only slightly differed in socio-economic status.

Another limitation of the current study is the fact that the analyses might be confounded by other variables, not considered (e.g. school characteristics) and by residual confounding arising from considering dichotomized versions of the HRB. Furthermore the use of parent/caregiver proxy measures of the health related behaviours may also have led to a possible bias and misclassification because of parents/caregivers’ social desirability. This potential bias needs to be acknowledged as a limitation of the current study, as well as the use of BMI as weight status parameter. Waist circumference might be stronger related to the adiposity status, however waist circumference is a much less reliable measure [19] and does not allow to distinguish between normal weight and overweight/obese preschoolers.

Of interest, but beyond the scope of the present study, is the fact that 12% of the boys and 11% of the girls were found to be underweight. It is of interest to further explore if and how underweight should be targeted. Furthermore, longitudinal studies on the causal interference between HRB and weight status and the potential time lag in young children are still needed.

## Conclusion

While some associations between HRB, mainly dietary behaviours, and BMI or the likelihood of being overweight/obese could already be detected at preschool age, in general the associations were limited, indicating a potential time lag between HRB and weight status. As unhealthy behaviours per se may not be perceived as problematic, parents/caregivers have to be sensitized of the fact that HRB are important independently of the weight status of their child. The challenge lies in determining ways to effectively motivate and support parents and other caregivers of young children to optimize practices related to young children’s HRB.
